# A Casz1–NuRD complex regulates temporal identity transitions in neural progenitors

**DOI:** 10.1038/s41598-021-83395-7

**Published:** 2021-02-16

**Authors:** Pierre Mattar, Christine Jolicoeur, Thanh Dang, Sujay Shah, Brian S. Clark, Michel Cayouette

**Affiliations:** 1 Cellular Neurobiology Research Unit, Institut de Recherches Cliniques de Montréal (IRCM), Montreal, QC H2W 1R7 Canada; 2 John F. Hardesty, MD, Department of Ophthalmology and Visual Sciences, Washington University School of Medicine, St. Louis, MO 63110 USA; 3 Department of Developmental Biology, Washington University School of Medicine, St. Louis, MO 63110 USA; 4 Department of Anatomy and Cell Biology, and Division of Experimental Medicine, McGill University, Montreal, QC H3A 0G4 Canada; 5 Department of Medicine, Université de Montréal, Montreal, QC H3T 1J4 Canada; 6 Present Address: Department of Cell and Molecular Medicine, University of Ottawa, Ottawa, ON K1H 8M5 Canada; 7 Present Address: Ottawa Health Research Institute (OHRI), Ottawa, ON K1H 8L6 Canada

**Keywords:** Neurogenesis, Developmental neurogenesis, Epigenetics, Proteomics, Neural stem cells, Gliogenesis, Retina

## Abstract

Neural progenitor cells undergo identity transitions during development to ensure the generation different types of neurons and glia in the correct sequence and proportions. A number of temporal identity factors that control these transitions in progenitor competence have been identified, but the molecular mechanisms underlying their function remain unclear. Here, we asked how *Casz1*, the mammalian orthologue of *Drosophila castor*, regulates competence during retinal development. We show that Casz1 is required to control the transition between neurogenesis and gliogenesis. Using BioID proteomics, we reveal that Casz1 interacts with the nucleosome remodeling and deacetylase (NuRD) complex in retinal cells. Finally, we show that both the NuRD and the polycomb repressor complexes are required for Casz1 to promote the rod fate and suppress gliogenesis. As additional temporal identity factors have been found to interact with the NuRD complex in other contexts, we propose that these factors might act through this common biochemical process to regulate neurogenesis.

## Introduction

During neurogenesis, virtually all neural stem and progenitor cells change their output dynamically over developmental time, first generating neurons, and then switching to generate glia^1^. In regions of the central nervous system (CNS) such as the neocortex and retina, progenitors undergo additional temporal transitions in competence to generate specific neuron subtypes at precise stages of development. In the vertebrate retina, retinal progenitor cells (RPCs) have two distinctive phases of multipotency during which they produce different neuronal subtypes and glia^2–5^. While many developmental regulators of retinal cell fate specification have been described, few determinants of RPC competence have been identified^6–9^. It thus remains largely unclear how transitions between vertebrate competence states are orchestrated.

While the molecular mechanisms that control temporal identity in vertebrates are poorly understood, transcription factor cascades controlling this process in *Drosophila* have been identified^10^. In the fly ventral nerve cord, most neural stem cells express a sequence of transcription factors as development proceeds, namely: hunchback, Krüppel, nub/pdm2 (collectively pdm), castor, and grainyhead^10^. This cascade of ‘temporal identity factors’ acts as a general timing mechanism to coordinate the output of hundreds of individual neuroblast lineages within the *Drosophila* CNS, but how it integrates coherently into so many different lineages remains unclear at the mechanistic level.

To identify and characterize the molecular pathways involved in temporal transitions during neural development, we chose to focus on the zinc finger transcription factor *Casz1*, which is the murine orthologue of *Drosophila castor*. In fly neuroblasts, *castor* is amongst the most influential of the temporal factors^11^, and we have previously reported that *Casz1* plays a conserved role in regulating progenitor potential in the developing mouse retina^12^. We and others have previously shown that Casz1 interacts physically and functionally with key subunits of the polycomb repressive complex (PRC)^13,14^. Casz1 was additionally shown to associate with the nucleosome remodeling and deacetylase (NuRD) complex in cultured cells^15^, but whether PRC or NuRD mediate Casz1 functions in neurodevelopment had not previously been addressed. Here, we address this question in the retina using biochemical and genetic assays.

## Results

### *Casz1* suppresses Müller glia production in postnatal retinal progenitors

**Figure 1:**
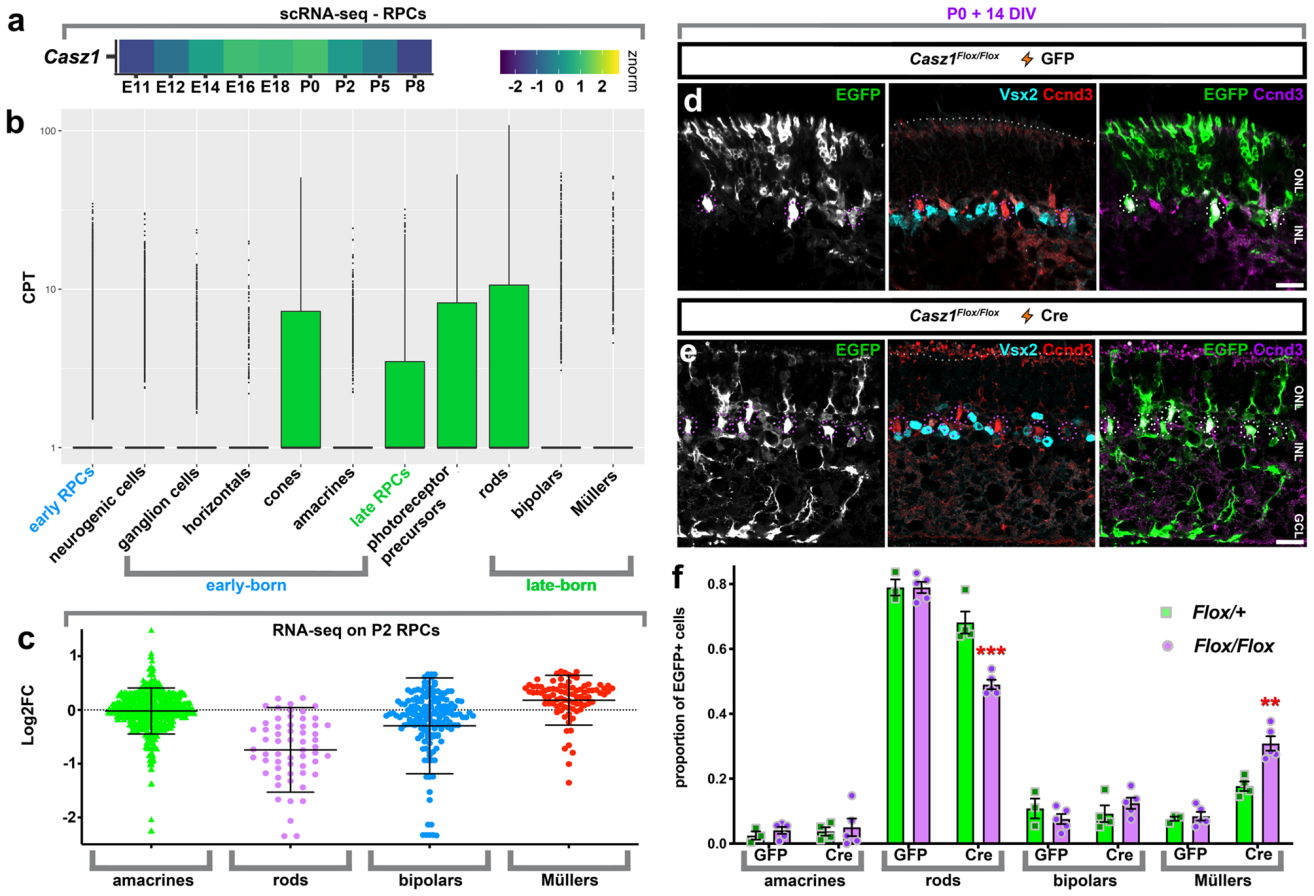
*Casz1* controls the rod versus Müller glia fate decision in postnatal retinal progenitors. (**a,b**) reanalysis of scRNAseq expression data^3^. (**a**) Normalized *Casz1* mRNA expression levels in RPCs during development. (**b**) Mean *Casz1* mRNA expression (counts per 10 000 transcripts: CPT). (**c**) Re-analysis of previously published RNA-seq data from sorted P2 *Casz1* cKO vs. control RPCs (n = 2)^13^. Plot depicts Log_2_ fold-change in gene expression for cell subtype marker genes based on published cell-type -specific profiling^63, 64^. (**d,e**) P0 *Casz1*^*Flox/Flox*^ or *Casz1*^*Flox/*+^ retinal progenitors were transfected with GFP control (**d**) or Cre (**e**) constructs, and cultured ex vivo for 2 weeks to allow development to reach completion. Explants were then harvested, sectioned, and stained for Ccnd3 and Vsx2 proteins, which mark Müller glia and bipolar cells, respectively. Scale bar = 20 microns. (**f**) Quantitation of the cell-type composition of the resultant transfections. See Table S1 for statistical summary. **p < 0.01; ***p < 0.001. *ONL* outer nuclear layer; *INL* inner nuclear layer; *GCL* ganglion cell layer.

The expression of *Casz1* mRNA and protein is low in the early retina, but increases progressively as development proceeds^12,16^. We took advantage of a previously published gene expression atlas of retinal development^3^ to re-examine *Casz1* expression at single cell resolution. We first confirmed that relative expression levels of *Casz1* steadily increase in RPCs, peaking at around P0, and declining postnatally (Fig. 1a). Within annotated cell types, although *Casz1* levels were higher in late RPCs, mRNA expression levels decreased as cells committed to bipolar or Müller fates, whereas levels increased considerably as cells committed to the photoreceptor trajectory and differentiated into rods (Fig. 1b).

Using a *Casz1* conditional knockout (cKO) mouse, we previously reported that *Casz1* is required in the retina to suppress the production of early-born neurons and late-born Müller glia, while promoting rod photoreceptors^12^. Accordingly, analysis of previously published RNA-seq data comparing sorted P2 RPCs^17^ revealed that expression of rod marker genes was systematically reduced in *Casz1* cKOs, whereas Müller marker genes were increased (Fig. 1c; Supplemental Data File 1). Thus, Casz1 biases RPCs to systematically promote the expression of rod photoreceptor genes and inhibit Müller genes.

As our previous work inactivated *Casz1* in early-stage RPCs, the specific requirement for *Casz1* in perinatal RPCs remained unclear. To address this gap, we introduced GFP control (pCIG2) or Cre recombinase together with GFP (pCIG2 + pCag-Cre) acutely into cohorts of P0 *Casz1*^*Flox/*+^ or *Casz1*^*Flox/Flox*^ RPCs using electroporation. We then cultured the transfected retinas ex vivo for 14 days, after which retinogenesis is complete. Cell types are born in very similar proportions in organotypic culture versus in vivo, although ganglion cells degenerate^18^. No significant difference in cell production was observed in GFP transfections from either genotype. However, when Cre was transfected, rod photoreceptor production was significantly reduced in *Casz1*^*Flox/Flox*^ explants (Fig. 1d–f; Table S1). This reduction was accompanied by a ~ fourfold increase in Müller glia. We conclude that in RPCs, *Casz1* expression levels rise and fall in synchrony with the developmental kinetics of rod production, and that *Casz1* is functionally required in postnatal RPCs to promote rods and suppress Müller glia production.

### *Casz1* interacts with the NuRD complex in RPCs

**Figure 2:**
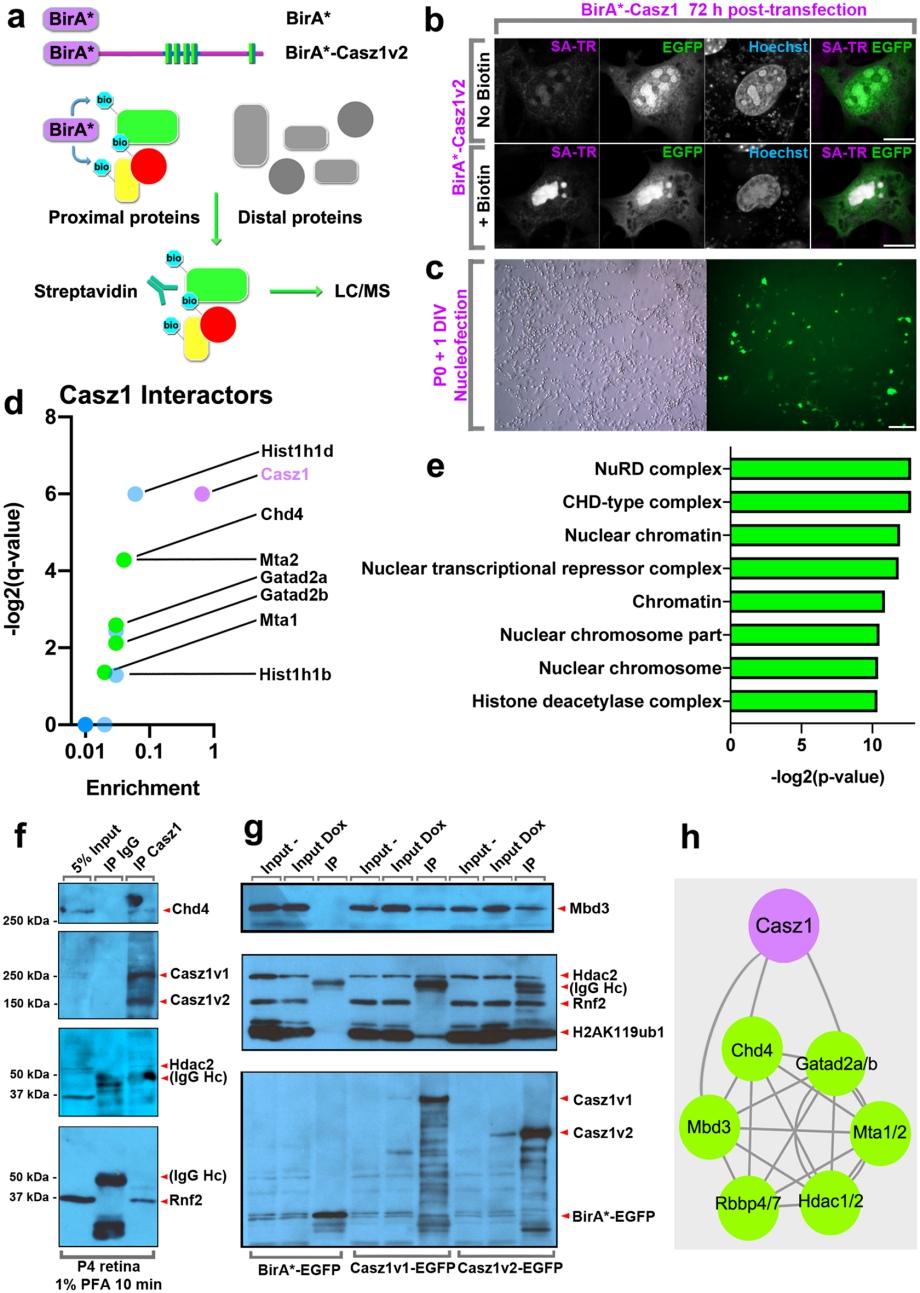
BioID proteomics identifies Casz1 interacting proteins from cultured retinal progenitors. (**a**) BioID strategy. Proteins are tagged with a mutant version of the bacterial biotin ligase BirA (BirAR118G, also called BirA*). BirA* promiscuously biotinylates all proteins with a free primary amine group (i.e. lysine residues). Biotinylated proteins are isolated using streptavidin coupled beads and subjected to liquid chromatography, tandem mass spectrometry (LC/MS–MS). (**b**) Representative images of the nuclear localization of BirA*-tagged Casz1v2 constructs transfected into 3T3 fibroblasts and cultured with or without exogenous biotin. Biotinylated proteins are visualized using Texas Red-conjugated streptavidin (SA-TR). Scale bar = 10 microns. (**c**) Transfection of BioID constructs into dissociated retinal progenitor cultures using nucleofection. Scale bar = 50 microns. (**d**) Volcano plot showing enrichment and statistical signficance (q-value) of Casz1 interactors (n = 10) versus control (n = 7) as determined via the Benjamini, Krieger and Yekutieli 2-stage linear step-up procedure for false-discovery correction. Core NuRD complex proteins are shown in green. Other proteins are shown in blue. Casz1 is shown in purple. (**e**) GO terms analysis of enriched annotations for statistically significant Casz1 interactors via ShinyGO^59^. (**f**) Validation of Casz1/NuRD complex interactions using cross-linking assisted immunoprecipitation from P4 retinas. (**g**) Validation of Casz1/NuRD complex interactions using stably expressing, doxycyline-inducible 293 T-REx cell lines. EGFP-fused control or Casz1 protein complexes were immunoprecipitated using anti-GFP antibodies. (**h**) Model of Casz1 interactome. Full-length scans of western data are presented in Fig. S4.

We reasoned that we could take advantage of the quantitatively large number of RPCs in perinatal retinas to identify Casz1 co-factors via biochemical purification. Unfortunately, we found that Casz1 protein could not be purified using standard methods. We could only detect full-length Casz1 isoforms when protein lysates were stabilized by formaldehyde cross-linking^13^. We thus chose to use BioID proteomics, as this approach bypasses the requirement to purify target proteins directly. Instead, the target protein is tagged with a BirA biotin ligase domain (BirA*) containing a mutation that abolishes target selectivity. BioID relies on the preferential biotinylation of the interactome as a function of molecular proximity (Fig. 2a,b). We focused on Casz1v2 since our previous experiments indicated that this variant promoted rod production^12^.

Next, control (pDEST-pcDNA5-FLAG-NLS-BirA) or Casz1v2 BioID constructs were introduced into primary retinal cultures by nucleofection. Transfected retinal cells were cultured using a defined serum-free medium^19,20^ (Fig. 2c). Transfection efficiencies were typically around 25%. Cultures were supplemented with biotin for 6 h and harvested. Since Casz1 is a nuclear protein in RPCs (Fig. S1a), we additionally extracted nuclei. Casz1 had previously been observed to interact with components of PRC1, both in cultured cells and in adult retinas^13,14^. To our surprise, when we examined the BioID interactome, we saw limited association between Casz1 and the PRC in perinatal retina, although Polycomb-associated proteins such as Cbx1/3/5, and Jarid2 were detected exclusively in the Casz1 interactome (Supplemental datafile 2).

Several core NuRD subunits were signficantly enriched in the Casz1 interactome, including Chd4, Gatad2a/b, and Mta1/2 (Fig. 2d). No NuRD proteins were ever observed in BioID complexes purified from control transfections (Supplemental datafile 2). Signficantly enriched proteins were subjected to Gene Ontology analysis. “NuRD complex” and “CHD-type complex” were the top-ranked terms, followed closely by several related terms (Fig. 2e; Table S2). GO terms related to alternative protein complexes were not prominently enriched. “Nucleosome” was the next most prominent protein complex, ranked as the 20th most enriched GO term. This rank order was not affected when significance testing was ignored and the analysis was instead performed on the 50 most enriched proteins in the Casz1 interactome.

To validate the observed association between Casz1 and the NuRD complex, we first performed cross-linking assisted immunoprecipitation using the *Rapid Immunoprecipitation Mass spectrometry of Endogenous proteins* (RIME) workflow^21^, which we previously showed stabilizes the Casz1 protein^13^. As expected, Casz1 protein complexes purified from developing retinas contained both Chd4 and Hdac2, as well as the polycomb subunit Rnf2 (Fig. 2f). Next, we performed conventional co-immunoprecipitation experiments using 293 T-REx cell lines that were engineered to stably and inducibly express control, Casz1v1- or Casz1v2-EGFP fusion proteins at levels that approximate physiological expression^22^. NuRD complex members like Hdac2 and Mbd3 were associated with both Casz1v1 and Casz1v2 isoforms (Fig. 2g). In contrast, the PRC1 subunit Rnf2 and its associated histone mark H2AK119ub1 were pulled down by Casz1v2, but were reduced or absent in Casz1v1 coIPs, as we previously reported (Fig. 2g)^13^.

We next assessed the co-localization of Casz1, Chd4, and Hdac proteins in retinal progenitors using immunohistochemistry. We observed that Casz1 was expressed throughout the nucleus, but accumulated at heterochromatic foci, enriched for marks such as Cbx1 and H3K9me3 (Fig. S1a–c). When we performed immunohistochemistry for NuRD proteins such as Chd4 or Hdac2, we observed that both proteins co-localized with many of these foci (Fig. S1d,e). This suggests that Casz1 is enriched within a subset of “NuRD bodies”^23^ in RPCs. In summary, our data support the conclusion that that Casz1 interacts primarily with the canonical NuRD complex in retinal progenitors (Fig. 2h).

### *Casz1* requires the NuRD complex to promote rods and suppress gliogenesis

**Figure 3:**
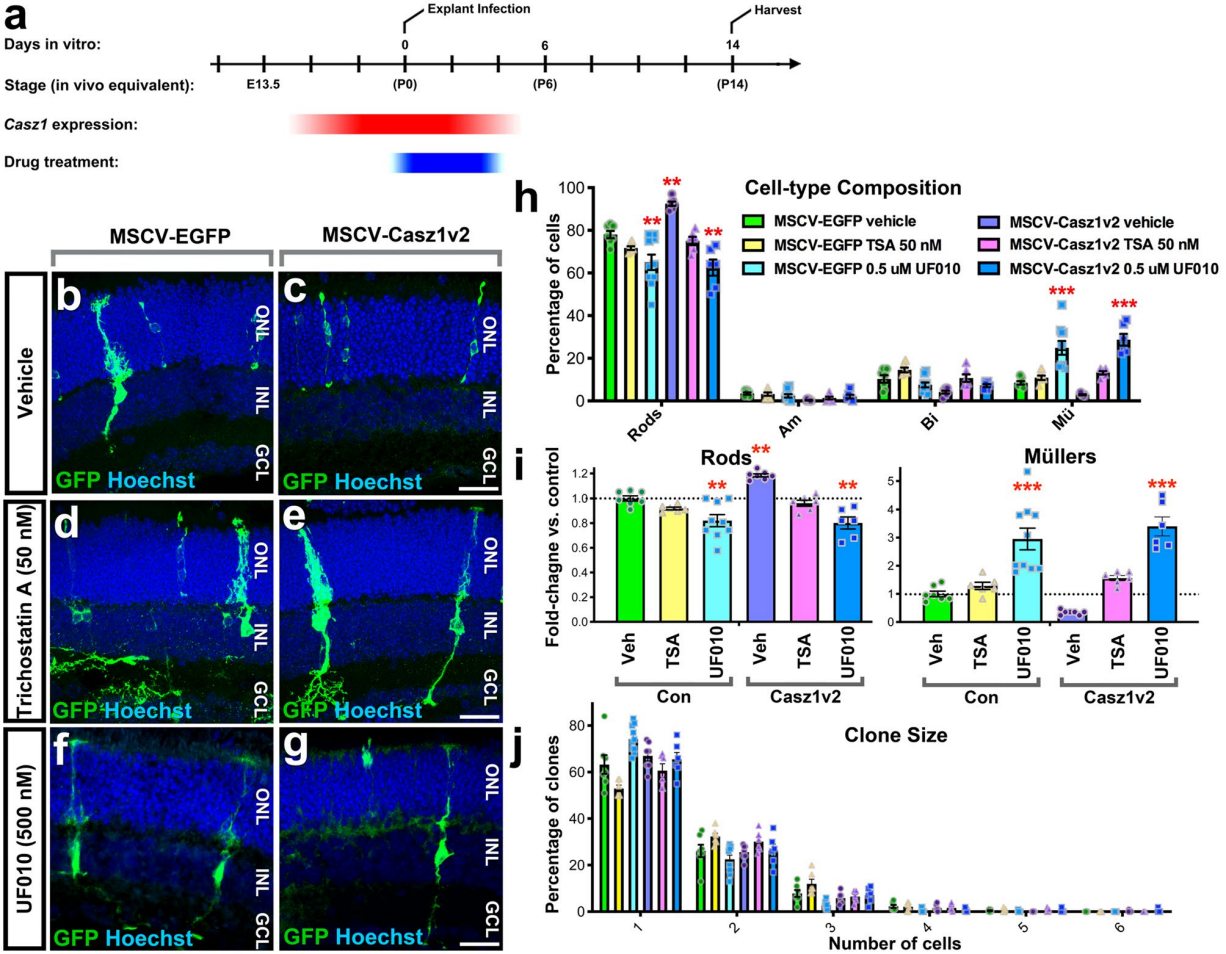
Casz1 requires histone deacetylase activity to control RPC output. (**a**) Experimental outline. Retroviruses were introduced at P0 at clonal density. Hdac inhibitors were added after 2–4 h, and left in place for an additional 4 days. Explants were harvested and analyzed after 14 days in vitro. (**b–g**) Examples of control or Casz1v2 transduced retroviral clones cultured in the presence of vehicle (**b,c**; control n = 7, Casz1v2 n = 9), 50 nM TSA (**d,e**; control n = 6, Casz1v2 n = 6), or 500 nM UF010 (**f,g**; control n = 6, Casz1v2 n = 8). Infected cells were stained with antibodies against GFP (green) and DNA was stained with Hoechst 33342 (blue). (**h,i**) Cell-type composition of retroviral clones. See Table S3 for statistical summary. **p < 0.01; ***p < 0.001. (**j**) Distribution of cells per clone. *ONL* outer nuclear layer; *INL* inner nuclear layer; *GCL* ganglion cell layer.

The NuRD complex has previously been shown to interact with Casz1 in cultured cell lines^15^, but the functional requirement for the NuRD complex in castor/Casz1-mediated neural cell fate decisions had not been addressed. NuRD possesses two enzymatic functions—nucleosome remodelling, and histone deacetylation. Interestingly, Hdac proteins had been shown to be required for retinal progenitor proliferation and rod photoreceptor gene expression^24–26^. To address whether Casz1 depends on Hdacs, we introduced control (pMSCV-EGFP) or Casz1v2-expressing retroviruses into P0 retinal progenitors. Retroviruses can only transduce dividing RPCs and were introduced at low titre, leading to single-copy, low-level transgene expression. After 2–4 h, we added vehicle, 50 nM trichostatin A (TSA)—a broad spectrum histone deacetylase inhibitor, or 500 nM UF010—a selective inhibitor of class I Hdacs, to the cultures for 4 days (Fig. 3a). Next, the drugs were washed out, and retinas were cultured ex vivo for 10 additional days to allow retinogenesis to reach completion. We then fixed the tissue explants and reconstructed the clones (Fig. 3b–g). Similar to our previous results with embryonic clones^12^, and conversely to what we observed by conditional ablation of *Casz1* at P0 (Fig. 1), Casz1v2 significantly increased the proportion of rods produced at the expense of Müller glia (Fig. 3h,i; Table S3). 50 nM TSA had no significant effect on rod vs. Müller production in control clones, but UF010 led to a significant reduction in rod production with a concomitant increase in Müller cells. When Casz1-transduced clones were treated with either TSA or UF010, Casz1v2-mediated rod production was lost, and Müller production increased significantly (Fig. 3h,i; Table S3). In agreement with our published analyses of *Casz1* loss- or gain-of-function in embryonic RPC lineages^12^, we found that clone size distributions were not significantly affected by either Casz1v2 or Hdac inhibition (Fig. 3i), indicating that differences in proliferation or apoptosis do not drive the observed changes in cell fate.

**Figure 4:**
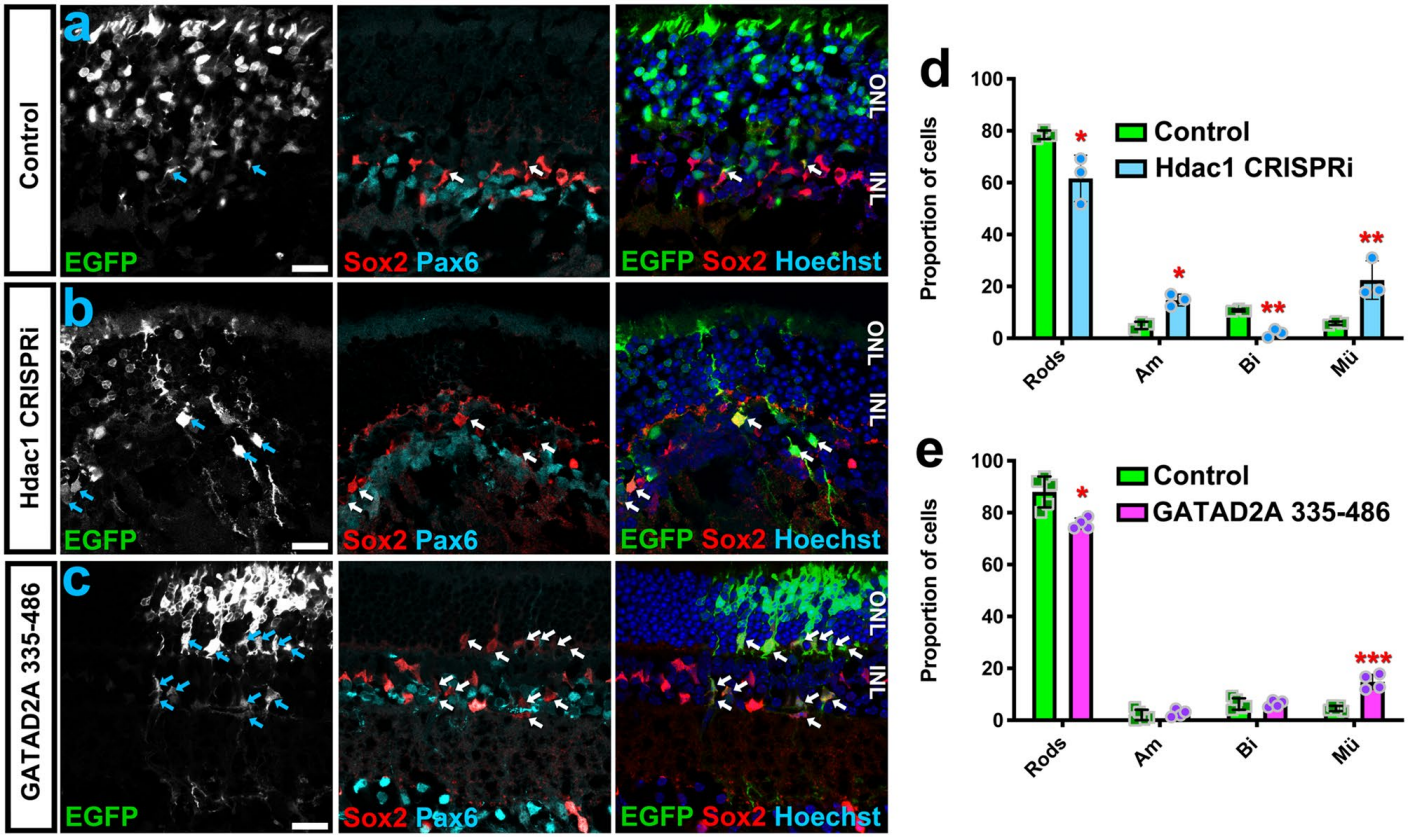
Hdac1 and NuRD regulate the rod versus Müller fate choice. (**a–c**) In vivo electroporation of P0 retinas with (**a**) Control (pCIG2 + dCas9-KRAB-MeCP2; n = 3), (**b**) Hdac1 CRISPRi (n = 3), or (**c**) GATAD2A-335-446 (n = 4). Retinas were harvested at P21 and stained for Sox2, Pax6, and DNA (Hoechst). Arrows indicate GFP + Sox2 + Müller glia, some of which are ectopically positioned in the ONL. Scale bars = 20 microns. (**d,e**) Quantitation of transfected cell fate. See Table S4 for statistical summary.

Previous investigations had demonstrated a requirement for Hdac1 in rod differentiation using drug inhibitors^26^. To provide genetic evidence, we used retinal electroporation to perform CRISPR interference (CRISPRi) on Hdac1 in vivo. First, the effect of CRISPRi on Hdac expression was validated in cell lines (Fig. S2). Then, retinas were co-transfected at P0 with constructs encoding GFP, dCas9-KRAB-MeCP2^27^, and a guide construct targeting the transcriptional start of Hdac1, or a control in which the guide construct was omitted. Retinas were harvested at P21, and the proportions of different transfected cell types were enumerated. As expected, we found that Hdac1 CRISPRi significantly reduced the proportion of rods and significantly increased the production of Müller glia versus control (Fig. 4a,b,d, Table S4).

While our experiments with pharmacological inhibitors demonstrated that Casz1 depends on Class I Hdacs, they did not conclusively demonstrate that Hdacs act via the NuRD complex. To manipulate the NuRD complex specifically, we overexpressed a C-terminal fragment of GATAD2A that was recently shown to sequester Chd4 and prevent its recruitment to the NuRD complex^28^. Overexpressing GATAD2A amino acids 335–446 via in vivo electroporation led to a significant reduction in rod production versus control (pCIG2), and a concomitant increase in Müller cells (Fig. 4c,e; Table S4). These data are consistent with the prediction that NuRD disruption would phenocopy *Casz1* loss-of-function. Taken together, our data demonstrate that both nucleosome remodelling and Hdac activities are required for the rod versus Müller fate choice.

### *Casz1* function depends on polycomb

**Figure 5:**
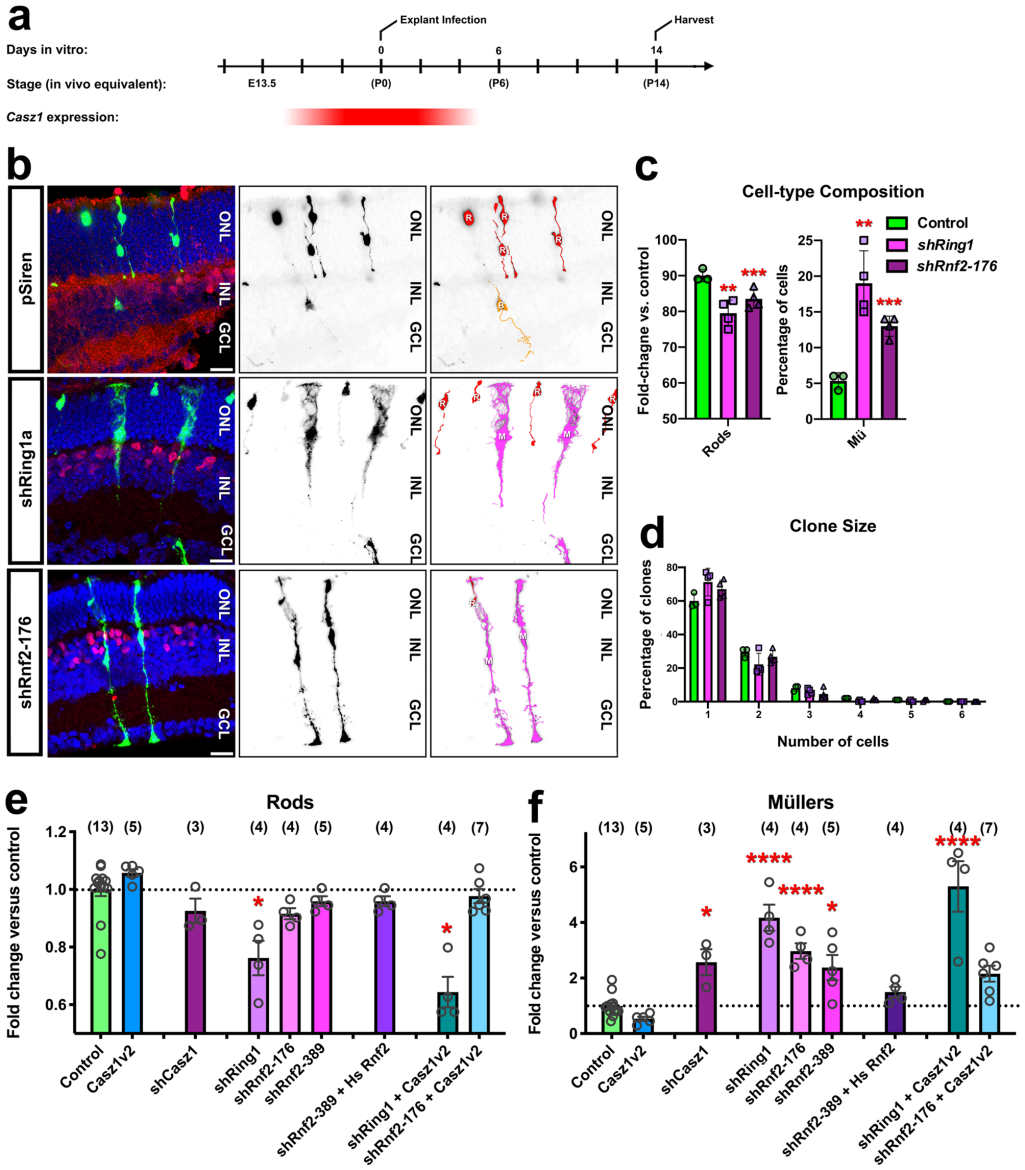
Casz1 requires polycomb to control retinal progenitor output. (**a**) Experimental timeline. (**b**) Examples of vector control (pSiren; n = 3) or shRNA clones (n = 4) that were transduced at P0 and cultured for 14 days in vitro, marked by ZsGreen expression (green). Sections were stained for Vsx2 (red) and Hoechst (blue). Scale bars = 20 microns. (**c,d**) Cell-type composition (**c**) and clone size distribution (**d**) of the resultant clones. See Table S5 for statistical summary. (**e,f**) Combinatorial expression/co-expression of Casz1 and polycomb loss or gain-of-function constructs. P0 retinas were electroporated with the indicated construct combinations, and harvested after 14 days in vitro. The proportion transfected rods (**e**) or Müller glia (**f**) was quantitated and normalized to the proportions obtained in control transfections. The control was pooled from overexpression (pCIG2: n = 8) and shRNA (pSiren: n = 5) vector transfections. The asterisks denote significant differences versus control as determined by one-way ANOVA with Tukey’s post-hoc test. n-values are presented in parentheses. *p < 0.05; **p < 0.01; ***p < 0.001; ****p < 0.0001. (**e**) Rod production in Casz1v2 transfections was significantly different versus Casz1v2 + shRing1 (p < 0.0001). (**f**) Müller glia production in Casz1v2 transfections was significantly different versus Casz1v2 + shRing1 (p < 0.0001) and Casz1v2 + shRnf2-176 (p < 0.05). See Table S6 for statistical summary.

Like temporal transcription factors, polycomb has been repeatedly implicated in developmental timing in neural progenitors^29–33^. Since our previous functional work suggested that Casz1v2 requires the PRC to safeguard transcription in rod photoreceptors, and biochemical work showed that Casz1 can associate with polycomb subunits (Fig. 2f,g)^13,14^, we addressed whether Casz1 might also depend on PRC1 function in addition to NuRD. We utilized retroviral shRNAs targeting Ring1 (Ring1a) or Rnf2 (Ring1b)^13^, which encode critical enzymatic subunits of PRC1. Similarly to *Casz1* loss-of-function, we found that shRNAs targeting Ring1 or Rnf2 significantly increased gliogenesis and reduced rod photoreceptor production versus control (pSiren-RetroQ-ZsGreen) when transduced into P0 RPCs ex vivo (Fig. 5a–c; Table S5). Clone size distributions were not significantly altered (Fig. 5d). Next, we performed combinatorial experiments, expressing Casz1v2 and shRNA combinations using explant electroporation. When Casz1v2 was co-expressed with Ring1 or Rnf2 shRNA constructs, Casz1v2-mediated suppression of Müller gliogenesis was reversed versus control (pooled from pCIG2 and pSiren-RetroQ-ZsGreen electroporations; Fig. 5e,f; Table S6; Fig. S3). These were specific effects, as they could be rescued by co-expressing a human *Rnf2* cDNA that could not be knocked down due to mismatches with the mouse shRnf2-389 hairpin^13^. We conclude that Casz1v2 requires PRC—likely downstream of the NuRD complex, to mediate its effects on cell fate.

## Discussion

Landmark work in *Drosophila* neuroblast lineages has shown that cascades of transcription factors act as molecular ‘clocks’ that progressively alter the developmental potential of progenitors. In contrast, epigenetic regulators were shown to be critical for shifting progenitor competence from neurogenesis to gliogenesis in mammalian lineages^29,34,35^. Whether temporal identity factors and epigenetic regulators cooperate to control temporal identity transitions remains unknown. Here we found that Casz1 interacts with the NuRD complex in retinal development—both physically and functionally. This suggests that epigenome remodelling might represent a general biochemical pathway used to regulate temporal competence in neural progenitors.

Precise control over cell proportions and birth order is critical for the establishment and fidelity of neural circuits. During development, the birth of rods and Müller glia peak asynchronously, with rod production peaking at P0/P1 and Müller production peaking at around P3/P4^36^. During development, Casz1 ontogeny in RPCs mirrors the rise and fall in rod production. Our functional data accordingly support the idea that Casz1 and the NuRD complex bias RPCs to favor perinatal rod production and to inhibit supernumerary and premature Müller production. While perinatal RPCs normally produce approximately 10 rods for each Müller glial cell, in *Casz1* cKO RPCs, this ratio falls to nearly 1:1. Conventional cell fate determinants are not well-suited to sculpt RPC output, as they typically drive cells out of the cell cycle, and are therefore not compatible with the RPC state. By contrast, Casz1 has no appreciable effect on RPC proliferation as determined by clone size analyses^12^. Our data suggest that Casz1 and the NuRD complex are important for biasing RPC output, and thereby control the shape of retinal lineages.

The NuRD complex has previously been implicated in neocortical development^37,38^, and human mutations in NuRD genes are associated with neurodevelopmental disorders and macrocephaly^39^. Previous studies also demonstrated that class I histone deacetylases such as Hdac1 and Hdac2 are required to promote rod photoreceptor differentiation^24–26^. Here, we used pharmacological inhibitors at lower doses that were closer to the IC50 values for class I enzymes^40^. This approach enabled us to separate the effects of Hdac inhibition on retinal cell fate specification from effects on the cell cycle or apoptosis, revealing that Hdacs are required for Casz1 functions. While our lower dosage regime for TSA did not reveal baseline effects on cell fate specification, our experiments with UF010 and Hdac1 CRISPRi both revealed significant decreases in rod photoreceptors and concomitant increases in Müller production that were quantitatively similar to the effects of *Casz1* abrogation. Effects on Müller gliogenesis were not directly measured in previous studies, however, Hdac inhibition was observed to promote the expression of Müller fate determination genes^25,26^. Our data thus suggest that Casz1 orchestrates the activity of Hdacs to regulate neurogenesis versus gliogenesis during retinal development.

Our results suggest a model where Casz1 interacts with the NuRD complex, which subsequently recruits PRC to the genome. Core NuRD proteins comprised 5 of the 8 proteins that were significantly enriched in the Casz1 BioID interactome. The remaining proteins were the linker histones Hist1h1b/d (H1.3 and H1.5), and histone 2B. Linker histones are key determinants of heterochromatin compaction, which agrees with the observed enrichment of Casz1 and NuRD proteins on the margins of chromocenters. While we detected little to no enrichment of other transcriptional regulatory complexes including polycomb, it remains possible that additional partner proteins may have been missed due to technical limitations of our BioID design, such as the fact that we focused on nuclear proteins, or due to placement of the BirA* fusion at the N-terminus of Casz1, which might have interfered with the labeling or association of certain partner proteins.

Physical and functional interactions between NuRD complexes and PRCs have been extensively described, albeit chiefly for PRC2^41–43^. Moreover, direct physical interactions between NuRD and PRC components have been previously observed in neural progenitors^41,44^. These studies suggested that NuRD/PRC cooperate to oppose gliogenesis. Since Casz1 is not expressed in neocortical progenitors, we propose that analogous transcriptional regulators orchestrate NuRD/polycomb recruitment in other regions of the developing CNS. Importantly, other studies have shown that the complexes are also required for cortical progenitors to undergo competence transitions and thereby promote gliogenesis^29,33^. Taken together, these experiments argue that NuRD and PRC cooperate to regulate developmental timing in the CNS, but that the specific activities of the complexes are context dependent.

In addition to Casz1, other temporal transcription factors have been previously been observed to associate with NuRD and PRC. Indeed, *Drosophila* dMi-2 (orthologous to Chd4) was originally discovered in a screen for proteins that interact with the temporal transcription factor hunchback. dMi-2 was shown to function in the polycomb pathway to repress *Hox* genes^45^. Similarly, in lymphocyte development, both NuRD and PRCs are associated with Ikzf1^46–48^, which was shown to confer early temporal identity in the developing mouse retina^49^. PRCs have also been shown to regulate competence transitions in *Drosophila* neuroblasts^32^. These observations suggest that temporal transcription factors might generally interact with NuRD/PRC to regulate competence transitions. While our previous work demonstrated that Casz1 acts to suppress the production of early-born retinal neurons^12^, the NuRD complex may have additional transcription factor partners at earlier stages of retinal development. It will thus be interesting to determine how the NuRD complex regulates the early competence of RPCs.

It was recently proposed that, in *Drosophila* neuroblasts, spatial factors would act on the epigenome to modify target gene access for the temporal factors, which would then generate different cell fates in a step-wise manner^10^. Our data suggest a model with reversed logic. We propose that temporal factor cascades utilize a common molecular pathway to perform step-wise modifications to the epigenome, which can then be read out by lineage-specific cell fate determinants—i.e. the spatial factors—to generate specific neuronal identities. We believe this model might explain how temporal transcription factor cascades can operate coherently in so many contexts, and why their sequential expression in neural progenitors has been conserved in evolution.

## Methods

### Animal care

Mouse husbandry was performed in accordance with the guidelines of the Canadian Council on Animal Care. Ethical protocols were approved by the IRCM, or the uOttawa (OHRI-2856; OHRI-2867) animal care committees. CD1 mice were obtained from Taconic or Charles River. *Casz1*^*Flox/Flox*^^12^ and *R26-Stop-EYFP*^50^ alleles and genotyping protocols were previously described. See also Table S7. Animals of either sex were used.

### Plasmids

Control and Casz1v2 retroviral (pMSCV-EGFP vector) and electroporation (pCIG2 vector) constructs were previously described^12^. shRNA and overexpression constructs for Casz1, Rnf2, and Ring1 were previously described^13^. pCag-Cre was was a gift from Connie Cepko (Harvard University; Addgene plasmid # 13775)^51^. dCas9-KRAB-MeCP2 was a gift from Alejandro Chavez and George Church (Harvard University; Addgene plasmid # 110821)^27^. The guide RNA construct was generated by excising Cas9 from pX330-U6-Chimeric_BB-CBh-hSpCas9 via restriction digest, which was a gift from Feng Zhang (Addgene plasmid # 42230)^52^. pDEST-pcDNA5-FLAG-NLS-BirA was a gift from Ann-Claude Gingras (Lunenfeld-Tanenbaum Research Institute)^53^. BirA*-Casz1v2 was generating by fusing Casz1v2 to the C-terminus of BirA* from plasmid pcDNA3.1 mycBioID, a gift of Kyle Roux (Addgene plasmid #35700)^54^. GATAD2A-335–446 was generated by subcloning a GATAD2A fragment containing an artificially introduced stop codon into vector pCIG2-NLSMT, which contains an SV40 nuclear localization sequence and 6xmyc epitope tags originally derived from plasmid pCS2 + MT^55,56^.

### Transfection, transduction, and culture

In vivo and ex vivo retinal electroporation were performed as described previously^12,13^. Retroviral preparation, ex vivo retroviral transduction, and clone reconstruction were performed as described previously^12,13,18,57^. Histone deacetylase inhibitors were administered 2–4 h after virus administration to ensure that viral integration was not altered. Trichostatin A was purchased from New England Biolabs (9950). UF010 was purchased from Cayman Chemical (21273). UF010 stocks were discarded after 2 weeks, and both drugs were stored frozen. In both cases, fresh drug was applied daily, and washed out after the 4th day. Stable lines were generated as described^22^, except that we expanded our cell lines from single cell clones. 1 µg/ml doxycycline (Alfa Aesar J60579) was added 24 h prior to harvest. P19 embryonal carcinoma cells and 3T3 fibroblasts were cultured and transfected as previously described^17,58^.

### Biochemistry

Crosslinking-assisted immunoprecipitation was performed according to the RIME workflow^21^. Protein immunoprecipitation was performed as described previously^13^. Antibodies are listed in Table S8.

### BioID proteomics

One P0 mouse litter (~ 20 eyes) was used for each experimental replicate. Retinas were dissected and dissociated as previously described^20^, and nucleofected with BioID constructs using an Amaxa Nucleofector II and the Mouse Neural Stem Cell Nucleofector Kit (Lonza; VPG-1004) according the manufacturer’s instructions. Cells were plated on poly-l-lysine/laminin coated dishes in RGM medium^19,20^, and cultured for 48 h prior to harvest. The culture medium was supplemented with exogenous biotin (50 μM final concentration) 6 h prior to harvest.

Cells were washed 3 × with PBS with 100 μM PMSF. Next, nuclei were harvested using cell lysis buffer (5 mM PIPES, pH 8.0, 85 mM KCL, 0.5% NP40, and Roche cOmplete, Mini, EDTA-free protease inhibitors). Cells were then scraped and collected, and the nuclei extracted for a further 15 min on ice with agitation. Lysis, sonication, bead coupling, and washing steps were performed as previously described^54^, using 60 μl of MyOne Streptavidin Dynabeads (Invitrogen).

Proteins were digested on-bead with 0.5 ug Sequencing Grade Modified Trypsin (Promega) overnight at 37 °C with agitation. The supernatants were collected and the beads were additionally washed two times with 100 μl ddH_2_0. Supernatants were pooled, reduced and alkylated with 9 mM dithiothreitol at 37 °C for 30 min followed by 17 mM iodoacetamide at room temperature for 20 min in the dark. Supernatants were acidified with trifluoroacetic acid and residual detergents removed using a Waters Oasis MCX 96-well Elution Plate following the manufacturer’s instructions. Samples were eluted in 10% ammonium hydroxide/90% methanol (v/v), dried, and reconstituted under agitation for 15 min in 15 µL of 5% FA.

### LC–MS/MS analysis

LC–MS/MS was performed using an LTQ Orbitrap Velos (ThermoFisher Scientific) equipped with a Proxeon nanoelectrospray Flex ion source as described^22^. Briefly, we used PicoFrit fused silica capillary columns (15 cm × 75 µm i.d; New Objective, Woburn, MA) with C-18 reverse-phase material and high pressure packing cells (Jupiter 5 µm particles, 300 Å pore size; Phenomenex, Torrance, CA). Data were acquired using a data-dependent top11 method combined with a dynamic exclusion window of 30 s. Protein samples were run in between blanks spiked with yeast ADH1 protein as a decoy to monitor and prevent spillover. Peptides mass fingerprinting was performed using Proteome Discoverer (version 2.1) and Mascot 2.5 (Matrix Science) against the Uniprot database. Bioinformatic analysis was performed using Scaffold (Version 4.11.0), using “Protein Threshold = 50%”; “Peptide Threshold = 80%”. To identify significantly enriched proteins, we filtered out all non-mouse proteins, and all proteins with an “extracellular region” GO term. We then selected all proteins with a “Cellular Component nucleus” GO term for further analysis. After filtering, the remaining 316 proteins were analyzed via multiple t-testing using the Benjamini, Krieger and Yekutieli 2-stage linear step-up procedure, with the false discovery rate set to 5%. Significantly enriched proteins were subjected to GO terms analysis using ShinyGO Version 0.61^59^. Proteomics data was deposited to the MassIVE repository (accession: MSV000085598).

### Histology and microscopy

Immunohistochemistry was performed as previously described^12,60^. Microscopy was performed using Zeiss LSM700, LSM710 (IRCM), or LSM880 instruments (CBIA Core, uOttawa) using Zen (Zeiss), Volocity (Perkin Elmer), Fiji (ImageJ), and Adobe Photoshop (Adobe) software. Primary antibodies are listed in Table S8. To quantitate cell types in explant electroporations, we used Ccnd3 to identify Müller glia, and Vsx2 to mark bipolars. Ccnd3-neg; Vsx2-neg INL cells were classified as amacrines. For in vivo electroporations, we stained tissue sections for Sox2 and Pax6. Within the INL, Pax6 + cells were classified as amacrine cells, while Sox2 + Pax6-neg cells were classified as Müller glia. Sox2-neg; Pax6-neg cells were classified as bipolars.

In retroviral experiments, we used cell morphology to classify cell types, but performed Vsx2 staining in order to assist discrimination between bipolars and amacrines. Cells were assigned to clones when GFP + cells occupied the same vertical column. Amacrines, bipolars, and Müller glia were all defined by the presence of a cell soma in the INL. Cells were classified as amacrines based on the absence of neurites projecting to the outer plexiform layer, whereas bipolar cells projected towards the outer plexiform layer. Cells were classified as Müller glia when they projected into the ONL. Cell counts were performed in explant regions where retroviral labelling was sparse, and where both the ONL and INL were intact. In all experiments, rods were identified by the presence of marker-negative nuclei in the ONL. All counts were performed manually and unblinded.

### RNA-seq analysis

*Casz1* transcript expression analyses within retinal progenitor cells (RPCs) across mouse retinal development and cell type enrichment were performed through re-analysis of the mouse single-cell RNA-sequencing dataset from GSE118614^3^. Heatmaps for RPC expression were generated based on a scaled (normalized z-score) mean expression of *Casz1* across RPCs at each time-point, with data normalized to cellular read-depth using transcript copies per 10,000 transcripts (CPT)^61^. Bar graphs assessing cell type enrichment of *Casz1* were generated in a similar manner, but instead examined a scaled enrichment of *Casz1* CPT across annotated cell types across all developmental ages profiled. Analyses were performed using Monocle 2.99.3^62^.

RNA-seq on control and *Casz1* cKO RPCs was previously published (GSE115778)^17^. To examine cell-type-specific gene expression, we adapted scRNA-seq cluster data^63^, selecting genes with “MyDiff” scores greater than 1.4. For rod photoreceptor genes, we supplemented the list with additional rod genes from a previous study^64^.

### Quantitation and statistical analysis

Densitometry measurements were performed using Adobe Photoshop CS5. Statistical analyses were performed using GraphPad Prism software. All tests were 2-tailed. n-values refer to biological replicates (different animals). All bar graphs display mean ± SEM. Sample sizes were not predetermined by power calculations.

## Supplementary Information

Supplementary Information 1.

Supplementary Information 2.

Supplementary Information 3.
