# Supplementary material for: A Casz1–NuRD complex regulates temporal identity transitions in neural progenitors
Source: Sci Rep. 2021 Feb 16;11:3858. doi: 10.1038/s41598-021-83395-7 (PMC7886867; doi:10.1038/s41598-021-83395-7)
Supplement: Supplementary file 3 — Supplementary Information 3. [file 41598_2021_83395_MOESM3_ESM.pdf]

Supplementary Information

A Casz1 - NuRD complex regulates temporal identity transitions in neural progenitors

Pierre Mattar*[1,2] , Christine Jolicoeur[1], Thanh Dang[2], Sujay Shah[2], Brian S. Clark[3], Michel Cayouette*[1,4,5]

[1]Cellular Neurobiology Research Unit, Institut de recherches cliniques de Montréal (IRCM), Montreal, QC, H2W 1R7, Canada
[2]Current address: Department of Cell and Molecular Medicine, University of Ottawa, Ottawa, ON, K1H 8M5, and Ottawa Health Research Institute (OHRI), Ottawa, ON, K1H 8L6
[3]John F. Hardesty, MD, Department of Ophthalmology and Visual Sciences, Washington University School of Medicine, St. Louis, MO 63110, USA; Department of Developmental Biology, Washington University School of Medicine, St. Louis, MO 63110, USA.
[4]Department of Anatomy and Cell Biology, and Division of Experimental Medicine, McGill University, Montreal, QC, H3A 0G4, Canada
[5]Department of Medicine, Université de Montréal, Montreal, QC, H3T 1J4, Canada

* Correspondance to: pmattar@ohri.ca, michel.cayouette@ircm.qc.ca

Table S1: Statistical summary for data presented in Fig. 1d. Values refer to mean ± SEM. NS, not significant. P-values were determined using Student's t-test.

**Treatment**	**# Replicates**	**#Cells**	**% Am**	**% Rods**	**% Bi**	**% Mu**
Control Flox/+	**3**	**1055**	**2.51 ± 1.26**	**78.97 ± 2.48**	**10.85 ± 3.06**	**7.68 ± 0.67**
Control Flox/Flox	**5**	**968**	**4.08 ± 1.07**	**78.94 ± 1.73**	**8.44 ± 1.33**	**7.59 ± 1.54**
p-value			NS	NS	NS	NS
Cre Flox/+	**4**	**815**	**3.73 ± 1.27**	**68.15 ± 3.39**	**9.23 ± 2.56**	**17.71 ± 1.48**
Cre Flox/Flox	**5**	**960**	**5.02 ± 1.07**	**49.03 ± 1.45**	**12.45 ± 1.73**	**30.85 ± 2.22**
p-value			NS	0.0008	NS	0.0024

Table S2. Enriched Gene Ontology terms associated with significant Casz1 interactors (via ShinyGO).

Enrichment FDR	Proteins in list	Total Proteins	Functional Category	Proteins
**1.8807462742268e-13**	5	16	NuRD complex	MTA1 GATAD2A GATAD2B MTA2 CHD4
**1.8807462742268e-13**	5	16	CHD-type complex	MTA1 GATAD2A GATAD2B MTA2 CHD4
**1.14340386492051e-12**	8	411	Nuclear chromatin	MTA1 GATAD2A GATAD2B HIST1H1D HIST1H1B MTA2 HIST1H2BA CHD4
**1.41404754620054e-12**	5	26	Nuclear transcriptional repressor complex	MTA1 GATAD2A GATAD2B MTA2 CHD4
**1.32402638175635e-11**	8	594	Chromatin	MTA1 GATAD2A GATAD2B HIST1H1D HIST1H1B MTA2 HIST1H2BA CHD4
**3.1491552083781e-11**	8	677	Nuclear chromosome part	MTA1 GATAD2A GATAD2B HIST1H1D HIST1H1B MTA2 HIST1H2BA CHD4
**3.99720787629483e-11**	8	711	Nuclear chromosome	MTA1 GATAD2A GATAD2B HIST1H1D HIST1H1B MTA2 HIST1H2BA CHD4
**4.47966597363735e-11**	5	57	Histone deacetylase complex	MTA1 GATAD2A GATAD2B MTA2 CHD4
**2.05052414406962e-10**	5	79	Transcriptional repressor complex	MTA1 GATAD2A GATAD2B MTA2 CHD4
**2.05052414406962e-10**	5	80	SWI/SNF superfamily-type complex	MTA1 GATAD2A GATAD2B MTA2 CHD4
**5.61253636773188e-10**	8	1048	Chromosomal part	MTA1 GATAD2A GATAD2B HIST1H1D HIST1H1B MTA2 HIST1H2BA CHD4
**5.61253636773188e-10**	5	101	ATPase complex	MTA1 GATAD2A GATAD2B MTA2 CHD4
**1.04776986253554e-09**	8	1156	Chromosome	MTA1 GATAD2A GATAD2B HIST1H1D HIST1H1B MTA2 HIST1H2BA CHD4
**2.68769963443815e-07**	9	3804	Nuclear lumen	MTA1 GATAD2A GATAD2B HIST1H1D HIST1H1B MTA2 HIST1H2BA CHD4 CASZ1
**6.60279538008743e-07**	9	4322	Membrane-enclosed lumen	MTA1 GATAD2A GATAD2B HIST1H1D HIST1H1B MTA2 HIST1H2BA CHD4 CASZ1
**6.60279538008743e-07**	9	4322	Organelle lumen	MTA1 GATAD2A GATAD2B HIST1H1D HIST1H1B MTA2 HIST1H2BA CHD4 CASZ1
**6.60279538008743e-07**	9	4288	Nuclear part	MTA1 GATAD2A GATAD2B HIST1H1D HIST1H1B MTA2 HIST1H2BA CHD4 CASZ1
**6.60279538008743e-07**	9	4321	Intracellular organelle lumen	MTA1 GATAD2A GATAD2B HIST1H1D HIST1H1B MTA2 HIST1H2BA CHD4 CASZ1
**1.82248658251272e-06**	4	219	Protein-DNA complex	HIST1H2BA CHD4 HIST1H1D HIST1H1B
**1.92523129289717e-05**	3	120	Nucleosome	HIST1H2BA HIST1H1D HIST1H1B
**1.92523129289717e-05**	8	4277	Non-membrane-bounded organelle	MTA1 GATAD2A GATAD2B HIST1H1D HIST1H1B MTA2 HIST1H2BA CHD4
**1.92523129289717e-05**	8	4262	Intracellular non-membrane-bounded organelle	MTA1 GATAD2A GATAD2B HIST1H1D HIST1H1B MTA2 HIST1H2BA CHD4
**2.08327137164028e-05**	3	128	DNA packaging complex	HIST1H2BA HIST1H1D HIST1H1B
**2.30327002371074e-05**	7	2900	Nucleoplasm	MTA1 GATAD2A GATAD2B MTA2 CHD4 CASZ1 HIST1H2BA
**3.58746799781305e-05**	5	1082	Nucleoplasm part	MTA1 GATAD2A GATAD2B MTA2 CHD4
**0.000110982809536114**	5	1379	Catalytic complex	MTA1 GATAD2A GATAD2B MTA2 CHD4
**0.0119790202570926**	2	391	Nuclear speck	GATAD2A GATAD2B
**0.0388913004026871**	2	744	Nuclear body	GATAD2A GATAD2B

Table S3: Statistical summary for data presented in Fig. 3h, i. Values refer to mean ± SEM. NS, not significant. P-values were determined using one-way ANOVA with Tukey's post-hoc test.

Treatment	# Replicates	#Clones	#Cells	% Am	% Rods	% Bi	% Mu
**MSCV-EGFP + vehicle**	**7**	**1128**	**1707**	**3.43 ± 0.53**	**78.00 ± 1.67**	**10.29 ± 1.74**	**8.43 ± 0.84**
**MSCV-EGFP + TSA**	**6**	**908**	**1503**	**3.17 ± 0.79**	**71.67 ± 0.88**	**14.50 ± 1.15**	**10.83 ± 1.08**
p-value vs. MSCV-EGFP veh				NS	NS	NS	NS
**MSCV-EGFP + UF010**	**6**	**1093**	**1424**	**2.30 ± 0.80**	**65.00 ± 3.60**	**7.50 ± 1.10**	**24.90 ± 3.30**
p-value vs. MSCV-EGFP veh				NS	< 0.05	NS	< 0.0001
**Casz1v2 + vehicle**	**9**	**1230**	**1736**	**0.29 ± 0.18**	**92.43 ± 1.00**	**4.14 ± 0.67**	**2.86 ± 0.26**
p-value vs. MSCV-EGFP veh				< 0.05	< 0.01	< 0.05	NS
**Casz1v2 + TSA**	**6**	**914**	**1348**	**1.33 ± 0.61**	**75.00 ± 1.93**	**10.66 ± 1.82**	**13.17 ± 0.79**
p-value vs. Casz1v2 veh				NS	< 0.01	< 0.05	< 0.05
**Casz1v2 + UF010**	**8**	**369**	**622**	**2.00 ± 0.89**	**62.50 ± 3.80**	**7.17 ± 0.75**	**28.67 ± 2.82**
p-value vs. Casz1v2 veh				NS	< 0.0001	NS	< 0.0001

Table S4: Statistical summary for data presented in Fig. 4. Values refer to mean ± SEM. NS, not significant. P-values were determined using Student's t-test.

Treatment	# Replicates	#Cells	% Am	% Rods	% Bi	% Mu
**Control** (pCIG2 + dCas9-KRAB-MeCP2)	**3**	**865**	**4.99 ± 0.82**	**78.47 ± 0.96**	**10.75 ± 0.26**	**5.79 ± 0.39**
**Hdac1 CRISPRi**	**3**	**621**	**14.74 ± 1.34**	**61.65 ± 5.18**	**1.97 ± 0.88**	**22.78 ± 4.59**
p-value (t-test)			0.0034	0.0331	0.0007	0.0180
**Control** (pCIG2)	**5**	**2405**	**1.92 ± 0.93**	**88.01 ± 2.66**	**6.27 ± 0.99**	**4.53 ± 0.44**
**GATAD2A-335-446**	**4**	**2685**	**2.71 ± 0.68**	**75.80 ± 1.07**	**6.34 ± 0.63**	**14.80 ± 1.46**
p-value (t-test)			NS	0.0062	NS	0.0001

Table S5: Statistical summary for data presented in Fig. 5c, d. Values refer to mean ± SEM. P-values were determined using one-way ANOVA with Dunnett's post-hoc test.

**Treatment**	**# Replicates**	**#Clones**	**#Cells**	**% Am**	**% Rods**	**% Bi**	**% Mu**
**Control (pSiren)**	**3**	**495**	**757**	**1.00 ± 0**	**90 ± 1.00**	**3.33 ± 0.33**	**5.33 ± 0.67**
**shRing1a**	**4**	**800**	**1082**	**0.75 ± 0.25**	**79.50 ± 1.84**	**1.25 ± 0.25**	**19.00 ± 2.27**
p-value vs. control				NS	0.0172	0.0112	0.0006
**shRnf2-176**	**4**	**754**	**1067**	**0.50 ± 0.29**	**83.50 ± 1.32**	**2.50 ± 0.50**	**13.00 ± 0.71**
p-value vs. control				NS	0.0002	NS	0.0171

Table S6: Statistical summary for data presented in Fig. 5e, f. Values refer to mean ± SEM. P-values were determined using one-way ANOVA with Tukey's post-hoc test.

Treatment	# Replicates	#Cells	% Rods	% Mu
**Control (pCIG2 n=8; pSiren n=5)**	**13**	**4576**	**85.83 ± 2.01**	**6.66 ± 0.73**
**Casz1v2**	**5**	**886**	**90.70 ± 1.13**	**3.58 ± 0.51**
p-value vs. control			0.9726	0.9782
**shCasz1**	**3**	**1633**	**79.43 ± 6.82**	**17.22 ± 3.11**
p-value vs. control			0.9575	0.0729
**shRing1a**	**4**	**405**	**65.39 ± 5.12**	**27.92 ± 3.15**
p-value vs. control			0.0043	< 0.0001
**shRnf2-176**	**4**	**625**	**78.56 ± 1.67**	**19.91 ± 1.90**
p-value vs. control			0.8522	< 0.0001
**shRnf2-389**	**5**	**1500**	**76.81 ± 3.99**	**15.94 ± 3.04**
p-value vs. control			0.5452	0.0427
**shRnf2-389 + HsRnf2**	**4**	**447**	**82.27 ± 1.48**	**10.01 ± 1.30**
p-value vs. control			0.1987	0.9785
**shRing1 + Casz1v2**	**4**	**279**	**55.24 ± 4.54**	**35.51 ± 6.09**
p-value vs. control			< 0.0001	< 0.0001
p-value vs. Casz1v2			< 0.0001	< 0.0001
**shRnf2-176 + Casz1v2**	**6**	**465**	**83.80 ± 2.01**	**14.44 ± 1.93**
p-value vs. control			> 0.9999	0.1006
p-value vs. Casz1v2			0.9134	0.0362

Table S7: Oligonucleotide sequences.

Name	Purpose	Sequence
CasSALacZ-F	genotyping	5’-GAGGGAGGTTGGCAAGAGGTGA-3’
CasSALacZ-R	genotyping	5’-GGACCTGGTTGTCATGGAGGAGAA-3’
CasFlox-R	genotyping	5’-CTGGCCGATGGCTTTTCAGTACA-3’
Rosa-26 (1)	genotyping	5’-AAAGTCGCTCTGAGTTGTTAT-3’
Rosa-26 (2)	genotyping	5’-GCGAAGAGTTTGTCCTCAACC-3’
Rosa-26 (3)	genotyping	5’-GGAGCGGGAGAAATGGATATG-3’
Hdac1 TSS F	CRISPRi	5’-CACCGCGGCTATAGGTGATCCCGGGGG-3’
Hdac1 TSS R	CRISPRi	5’- AAACCCCCCGGGATCACCTATAGCCGC-3’
Hdac2 TSS F	CRISPRi	5’-CACCAGTCCGGAGGTGCGCACAAGCGG-3’
Hdac2 TSS R	CRISPRi	5’- AAACCCGCTTGTGCGCACCTCCGGACT-3’
GATAD2A 335-486 F	dominant negative	5’- GAATTCCCGATGGTCACCTCTGCCGAGTCTCCA-3’
GATAD2A 335-486 R	dominant negative	5’- GAATTCCTAGGCCTGTGCAGGGGCCGTGCC-3’
shRing1-F:	shRNA	5'_gatccGTGCACAGAATGCCAGCAAATTCAAGAGATTTGCTGGCATTCTGTGCATTTTTTgatatcg_3'
shRing1-R:	shRNA	5'_aattcgatatcAAAAAATGCACAGAATGCCAGCAAATCTCTTGAATTTGCTGGCATTCTGTGCACg_3'
shRnf2-176-F:	shRNA	5'_gatccAGAACACCATGACTACAAATTCAAGAGATTTGTAGTCATGGTGTTCTTTTTTTGATATCg-3'
shRnf2-176-R:	shRNA	5'_aattcGATATCAAAAAAAGAACACCATGACTACAAATCTCTTGAATTTGTAGTCATGGTGTTCTg-3'
shRnf2-389-F:	shRNA	5'_gatccGGATCAACAAACACAACAATTCAAGAGATTGTTGTGTTTGTTGATCCTTTTTTgatatcg_3'
shRnf2-389-R:	shRNA	5'_aattcgatatcAAAAAAGGATCAACAAACACAACAATCTCTTGAATTGTTGTGTTTGTTGATCCg_3'

Table S8: Primary antibodies and dilutions.

Antigen	Species	Dilution	Supplier	Application
Casz1	Rabbit	1:1000	Seth Blackshaw Lab C5-2	IP/Western
Casz1	Guinea pig	1:200	Johan Ericsson Lab Cst95	IHC
Cbx1	Rat	1:500	Abcam ab10811	IHC
Ccnd3	Mouse	1:100	Santa Cruz D-7	IHC
Chd4	Rabbit	1:1000	Abcam ab72418	IP/Western
Chd4	Rabbit	1:200	Active Motif 39289	IHC
Flag	Rabbit	1:1000	Cell Signaling 2368P	Western
GFP	Rabbit	1:1000	Molecular Probes A11122	IP/IHC
GFP	Mouse	1:1000	DSHB GFP-G1	IHC
H2AK119ub1	Rabbit	1:1000	Cell Signaling 8240	Western
H3K9me3	H3K9me3	1:200	Diagenode C15410056	IHC
Hdac1	Rabbit	1:200<br>1:1000	Bethyl A300-713	IHC<br>Western
Hdac2	Rabbit	1:200<br>1:1000	Bethyl A300-705	IHC<br>Western
Mbd3	Rabbit	1:1000	Bethyl A302-529	Western
Pax6	Mouse	1:10	DSHB Pax6	IHC
Rnf2	Mouse	1:1000	MBL D139-3	Western
Sox2	Goat	1:500	R&D Systems AF2018	IHC
Vsx2	Sheep	1:200	Exalpha Biologicals X1180P	IHC

Fig. S1. Casz1 and NuRD complex proteins localize to heterochromatin in retinal progenitors. (**a**) Casz1 immunohistochemistry on E17.5 *Casz1$^{Flox/Flox}$; α–Pax6::Cre; R26-Stop-EYFP* (cKO) retinal sections in mosaic regions containing recombinant (ie. EYFP+) and unrecombined (EYFP-negative) RPCs. Note that Casz1 protein staining disappears in the EYFP+ cells (arrowheads). Casz1 is localized diffusely throughout the nucleus, but is enriched on the outer margins of chromocenters (red circles). (**b, c**) Casz1 protein colocalizes with the heterochromatic marks H3K9me3 (**b**) or Cbx1 (**c**) on the margins of chromocenters (red circles) in E18.5 retinal progenitors. (**d, e**) Double-staining for Casz1 and Chd4 (**d**) or Hdac2 (**e**) on P0 wild-type RPCs. Arrows mark double-positive foci. Scale bars = 5 microns.

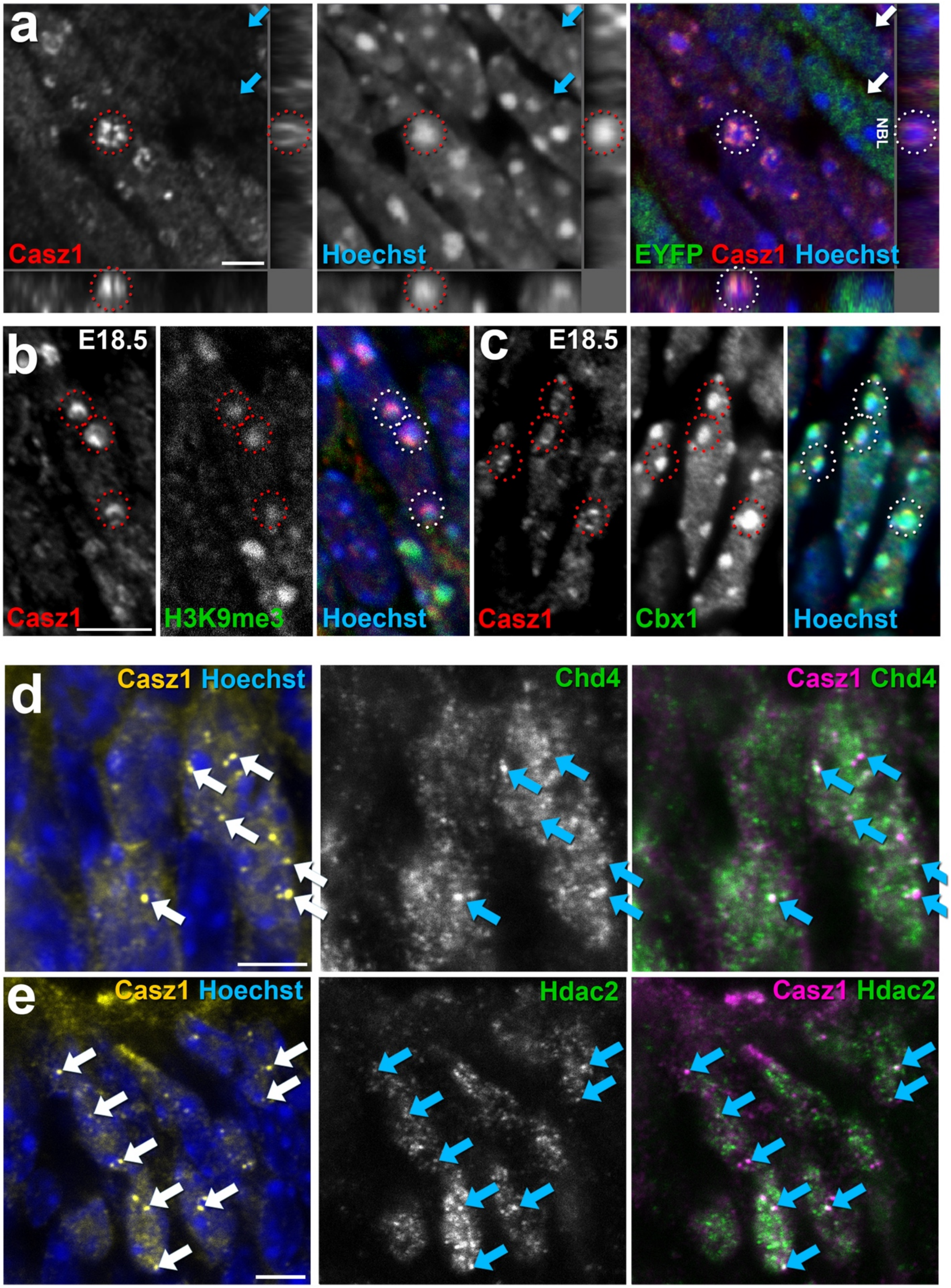

Mattar et al., Fig. S1

Fig. S2. Hdac1 CRISPRi validation. (**a, b)** Murine P19 embryonal carcinoma cells were transfected with GFP (pCIG2) and dCas9-KRAB-MeCP2, with or without guide constructs targeting the transcription start sites of Hdac1 or Hdac2 as indicated. (**a**) Cells were harvested and subjected to western blotting for Hdac1, GFP (reporting transfection efficiency), or ß-actin (loading control). (**b**) Densitometry was performed on Hdac1 protein levels normalized against ß-actin levels. * $p = 0.0171$, $n=5$. (**c-f**) *In vivo* electroporation of P0 retinas with Hdac1 CRISPRi. Retinas were harvested at P21 and visualized for GFP epifluorescence, Hdac1 immunohistochemistry (red) and Hoechst (blue) using Airyscan confocal microscopy. (**d, f**) GFP+ transfected cells or randomly-selected neighboring cells were analyzed for Hdac1 levels via densitometry. In both the ONL (**d, e**) and INL (**f, g**), transfected cells exhibited reduced Hdac1 levels in comparison to neighbors, although overall Hdac1 levels were lower in the ONL vs. INL. Scale bars = 10 microns.

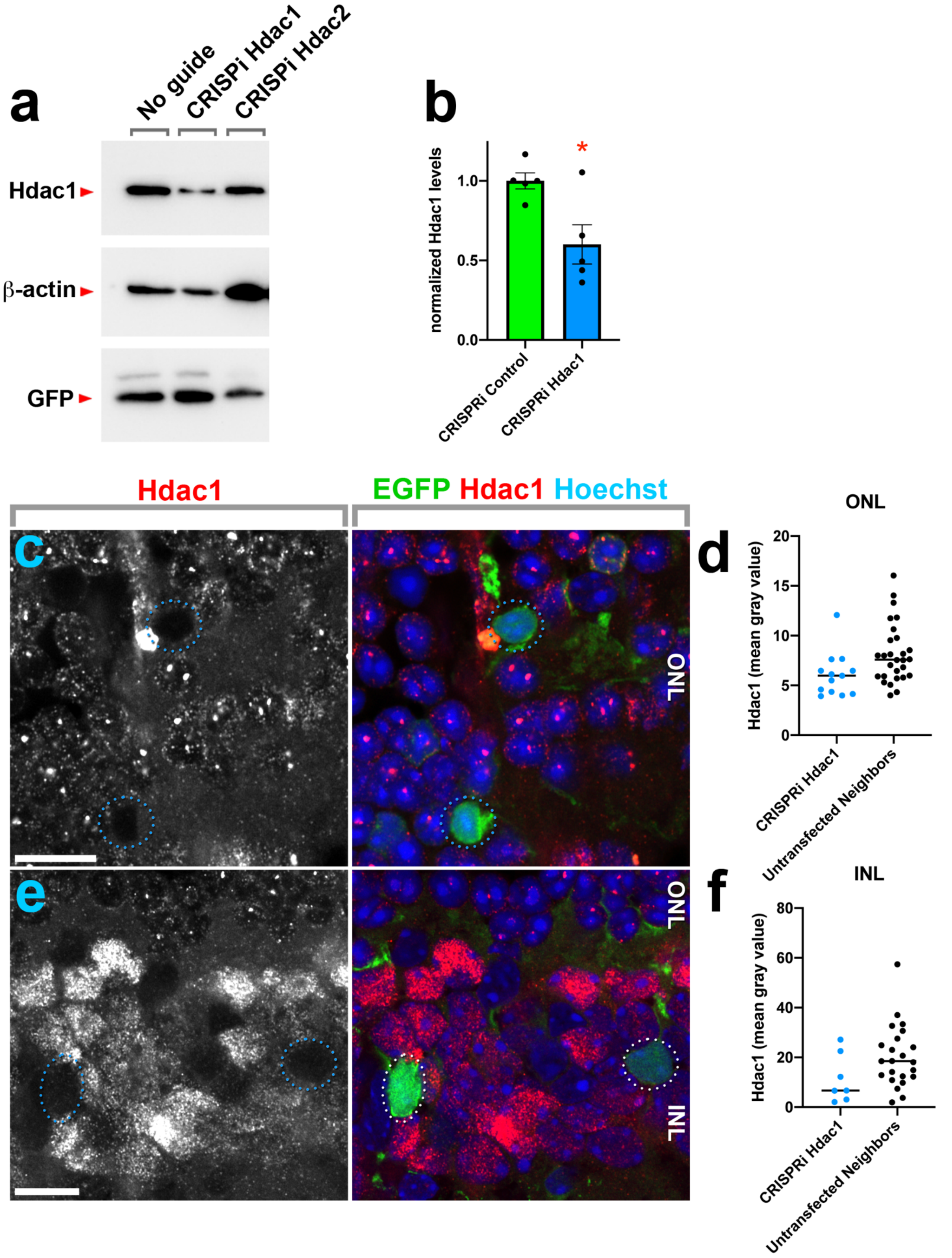

Mattar et al., Fig. S2

Fig. S3. Casz1 requires polycomb to control retinal progenitor output. (**a-d**) Representative examples of Casz1 and polycomb loss-of-function transfections. P0 retinas were electroporated with the indicated construct combinations, and harvested after 14 days *in vitro*. Explants were stained for Ccnd3, which marks Müller glia (circles). (**a**) Control (pCIG2); (**b**) Casz1v2; (**c**) shRnf2-176; (**d**) Casz1v2 + shRnf2-176. Dashed line indicates the boundary between the ONL and INL.

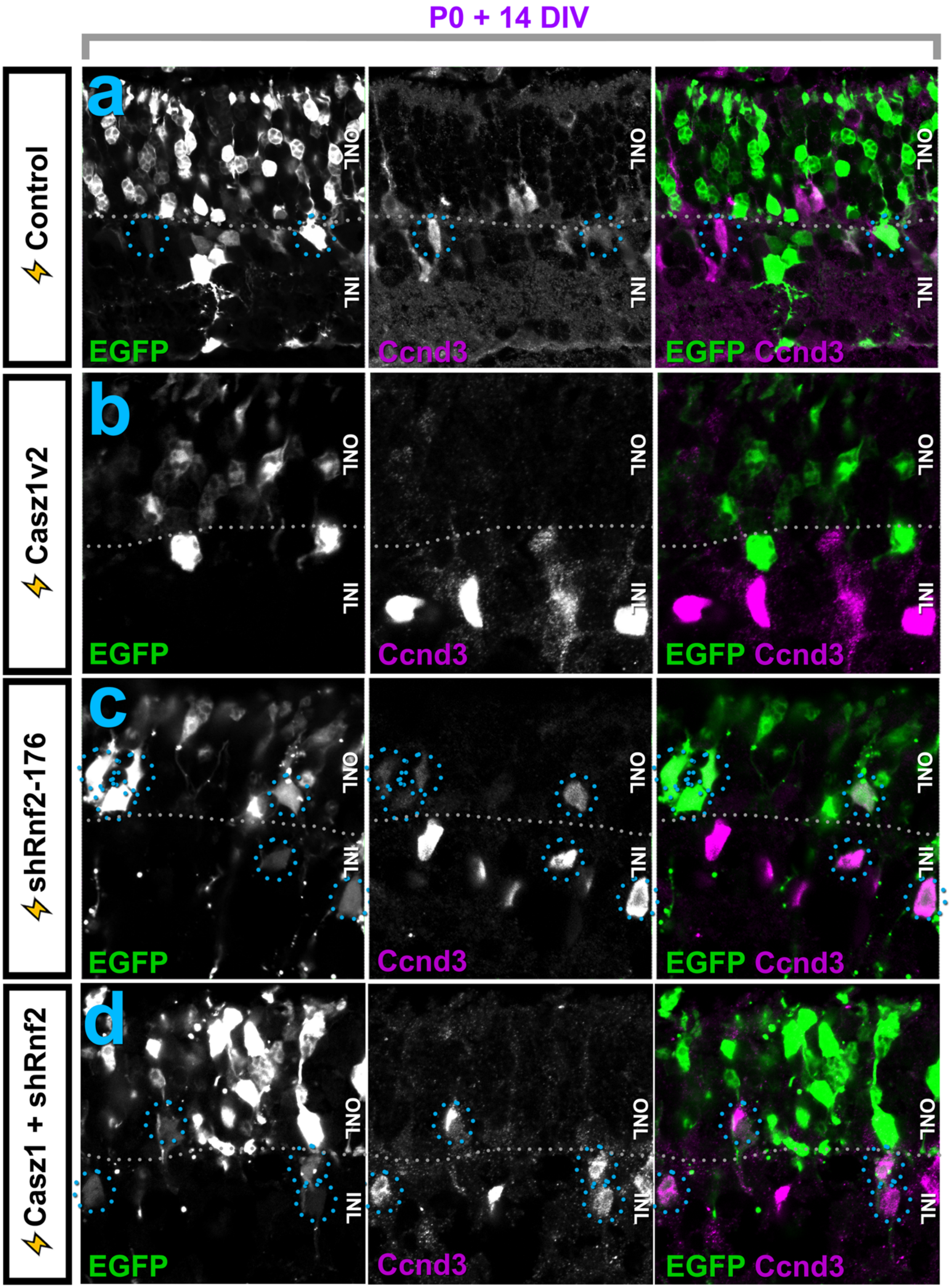

Mattar et al., Fig. S3

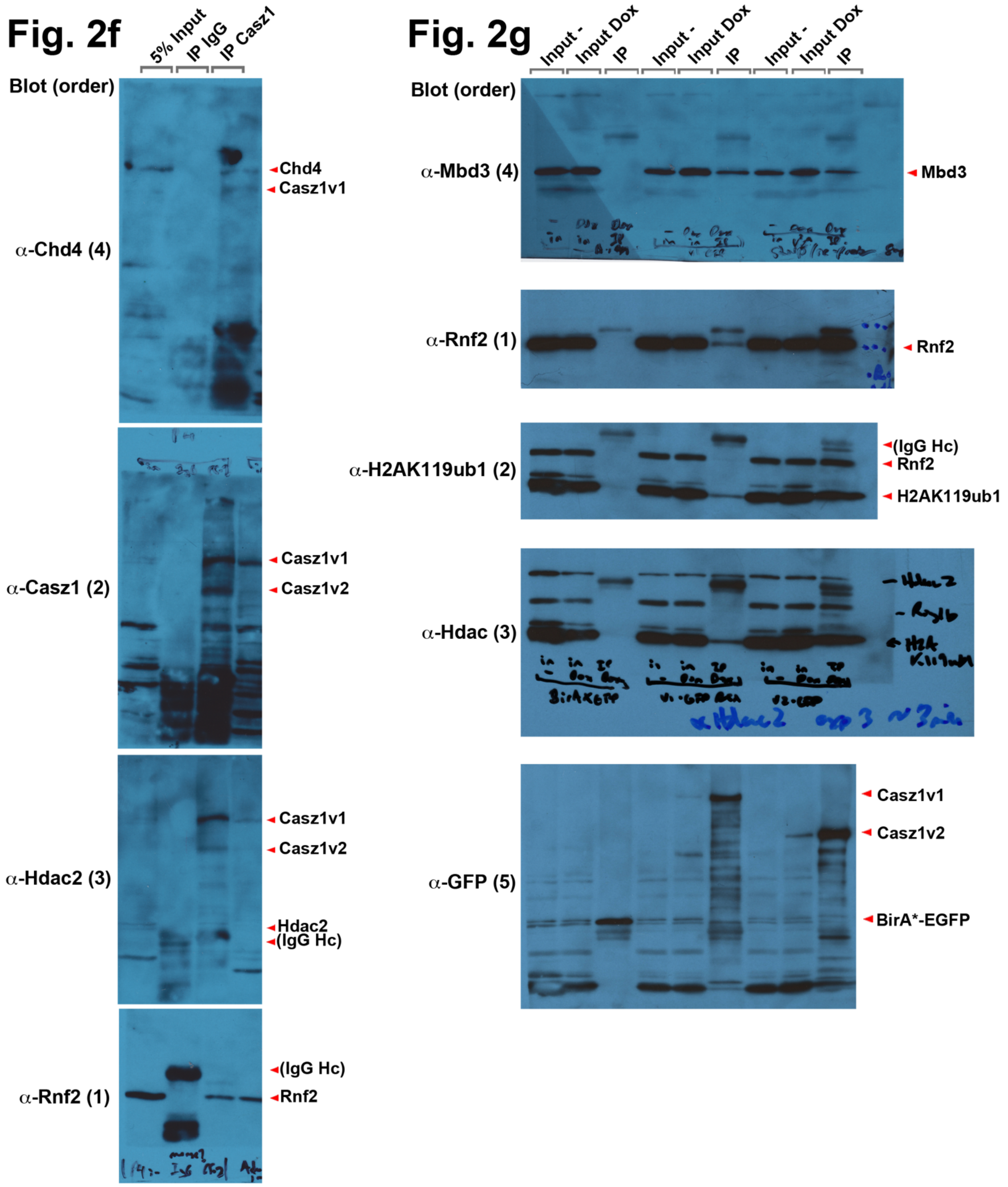

Fig. S4. Original scans of western data presented in Fig. 2.

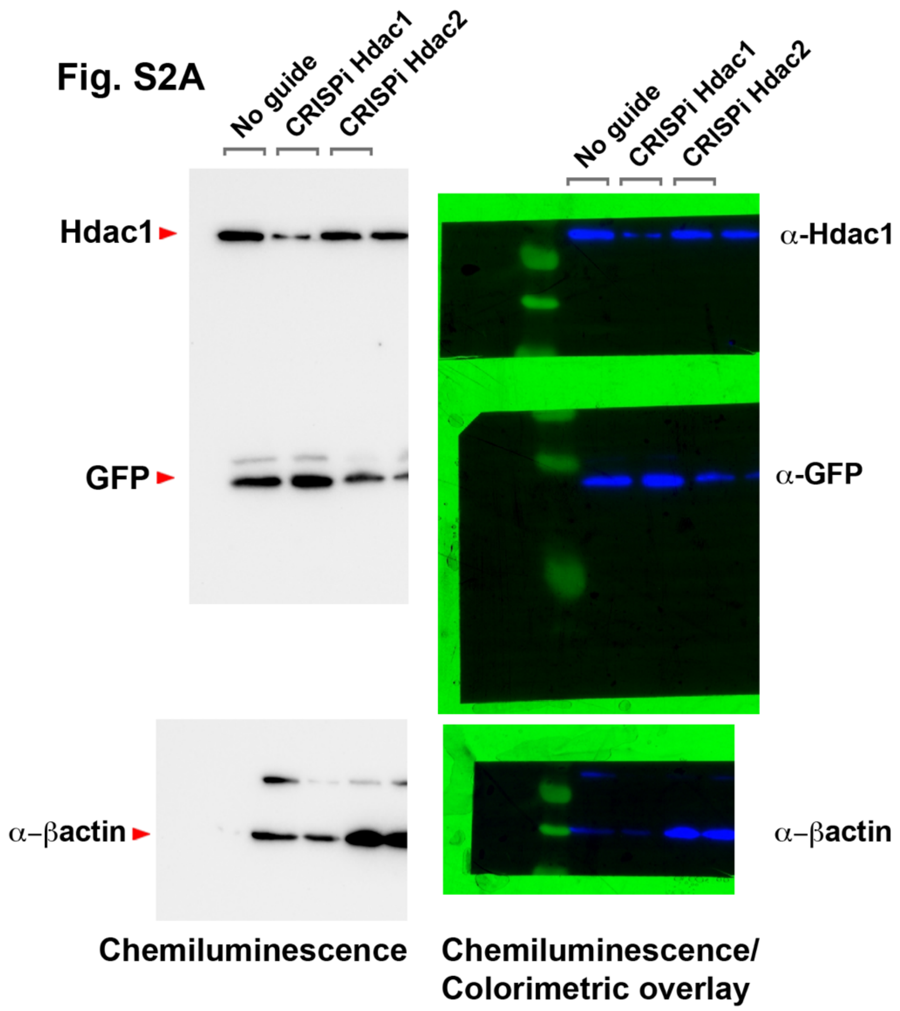

Fig. S5. Original scans of western data presented in Fig. S2.

Supplemental datafile 1. Spreadsheet of RNA-seq data presented in Fig. 1 (Excel file).

Supplemental datafile 2. Spreadsheet of BioID experimental results (Excel file).
